# Post-acne Erythema: A Systematic Review of Randomized Controlled Trials on the Comparative Efficacy and Safety of Interventions Across Clinical, Instrumental, and Patient-Reported Outcomes

**DOI:** 10.7759/cureus.116495

**Published:** 2026-09-18

**Authors:** Ahmed Alhussain, Mohammad Majrashi, Khalid Omar AlMubarak, Rayan Faleh Alqahtani, Nasir Abdullah Al Melhem, Shahad Saad S AlShammari, Thamer M Mubarah, Ghadeer A Alameer, Fatima Mohammed Al-Sawad, Hadeel Hesham Buali, Raghad Khalid Alenezi, Mohammed A Ababtain, Bader Ahmed Sabei, Abdulelah F Alshehri

**Affiliations:** 1 Medicine, Almaarefa University, Riyadh, SAU; 2 Neonatology, Riyadh Second Health Cluster, Riyadh, SAU; 3 Medicine, Imam Muhammad Ibn Saud Islamic University, Riyadh, SAU; 4 Family Medicine, King Faisal Specialist Hospital & Research Centre, Riyadh, SAU; 5 Medicine, Imam Abdulrahman Bin Faisal University, Dammam, SAU; 6 Medicine, King Faisal University, Al-Ahsa, SAU; 7 Medicine, King Saud bin Abdulaziz University for Health Sciences, Riyadh, SAU; 8 Clinical Research, Independent Researcher, Riyadh, SAU

**Keywords:** hyaluronic acid mesotherapy, intense pulsed light, laser therapy, post-acne erythema, systematic review

## Abstract

Post-acne erythema (PAE) is a persistent acne sequela without an established standard treatment. This systematic review evaluated the comparative efficacy and safety of interventions tested in randomized trials. PubMed/MEDLINE, Scopus, and Web of Science were searched from inception through August 1, 2026. Randomized parallel-group and split-face trials reporting erythema outcomes were eligible. Risk of bias was assessed with the Cochrane Risk of Bias 2 (RoB 2) tool. Heterogeneity precluded meta-analysis, and available findings were synthesized narratively. Nine trials involving 301 participants contributed 11 pairwise comparisons. Against untreated or blank controls, favorable effects were reported for picosecond alexandrite laser, narrow-spectrum vascular intense pulsed light (IPL), and non-cross-linked hyaluronic acid mesotherapy. A 577-nm semiconductor laser showed interim instrumental benefit, but blinded assessors found no final between-side difference. In active-treatment and add-on comparisons, narrow-spectrum IPL was favored over broad-spectrum IPL, neodymium-doped yttrium aluminum garnet (Nd) laser added to low-dose isotretinoin was favored over isotretinoin alone, and 577-nm pro-yellow laser was favored over topical brimonidine. Botulinum toxin type A was favored over broadband light at the earliest assessment only. Fractional laser and pulsed dye laser did not differ significantly, while delicate pulsed light and Q-switched 1064-nm Nd laser showed no sustained difference at the final assessment. Adverse events were mild and transient, although pigmentation and blistering occurred with one broad-spectrum IPL protocol. Five trials had some concerns, and four had high overall risk of bias. Trials suggested potential benefits, but small samples, heterogeneity, short follow-up, and reporting limitations precluded identification of a preferred treatment. Larger, prospectively registered trials using blinded assessment and standardized outcomes are needed.

## Introduction and background

Acne vulgaris is one of the most prevalent inflammatory skin disorders worldwide, affecting an estimated 9% of the global population across all age groups [1]. Its clinical burden may persist after active papules and pustules have resolved because visible changes frequently remain at previously inflamed sites. These residual manifestations are distinct from active acne and include pigmentary alterations, persistent erythema, and scarring. Post-acne erythema (PAE) is characterized by persistent erythematous macules occurring at the sites of healed inflammatory acne lesions [2].

The pathophysiology of PAE remains incompletely understood. Persistent redness is thought to reflect residual inflammation accompanied by dilation and proliferation of superficial dermal vessels and localized tissue edema [3]. Altered microvascular reactivity may also contribute to the persistence of erythema. Impaired epidermal barrier function, including reduced stratum corneum ceramide levels, has been demonstrated in acne-affected skin [4], although its specific role in maintaining PAE has not been established. PAE should be distinguished from post-inflammatory hyperpigmentation, which primarily results from increased melanin production and deposition [5], and from atrophic or hypertrophic scarring, which involves structural alteration of the dermis.

Because PAE most commonly affects the face, it is readily visible and may be difficult to conceal. Although some lesions gradually fade without intervention, erythema may persist sufficiently long to prompt repeated treatment and dermatologic consultation. Facial acne is associated with reduced self-esteem and impaired dermatology-related quality of life [6]. Persistent erythema may plausibly extend this burden beyond the resolution of active inflammatory lesions, but patient-reported evidence specifically addressing PAE remains limited. Its prevalence, natural course, and predictors of persistence have also not been established with precision.

Several therapeutic approaches have been investigated. Light- and laser-based interventions include vascular, fractional, and picosecond systems administered using heterogeneous wavelengths, fluences, pulse durations, treatment intervals, and numbers of sessions [7]. Pharmacological, injectable, and combination treatments have also been evaluated, reflecting the vascular, inflammatory, remodeling, and barrier-related mechanisms proposed for PAE. However, no intervention has been established as a universally accepted standard of care for this indication.

Interpretation of the available evidence is complicated by methodological and clinical heterogeneity. Individual trials are generally small, while comparators range from untreated contralateral facial sides to active interventions and background treatment alone. Treatment schedules, assessment times, and follow-up durations also vary considerably. In addition, the terms post-inflammatory erythema and PAE are sometimes applied to overlapping populations, and outcomes are measured using clinician-rated scales, instrumental erythema indices, colorimetry, and image-analysis systems that should not be assumed to be interchangeable. Split-face trials randomize facial sites within the same participant and require paired analytical and reporting methods that differ from those used in parallel-group trials [8]. Consequently, improvement within a treated group cannot independently establish comparative efficacy, and clinical, instrumental, patient-reported, and safety outcomes require distinct interpretation.

This systematic review aimed to identify and critically appraise randomized controlled trials (RCTs) evaluating interventions for PAE and to synthesize randomized comparative evidence on efficacy and safety across clinical, instrumental, and patient-reported outcomes. Attention was given to differences in study design, comparator selection, outcome measurement, assessment timing, and risk of bias.

## Review

Methods

Study Design and Reporting Standards

This systematic review evaluated RCTs of therapeutic interventions for PAE. Parallel-group and intraindividual split-face randomized designs were eligible. The review was reported in accordance with the Preferred Reporting Items for Systematic Reviews and Meta-Analyses (PRISMA) 2020 statement [9]. The review protocol was not prospectively registered.

Literature Search Strategy

PubMed/MEDLINE, Scopus, and Web of Science were searched from database inception through August 1, 2026. The search combined terms describing erythema persisting after acne with terms identifying randomized and split-face trials. Terminological variants were included because the condition has been described as PAE, post-inflammatory erythema following acne, or acne erythema. The search was not restricted by intervention type. Reference lists of included studies and relevant reviews were screened manually. No language restrictions were applied. Duplicate records were removed before screening. Search syntax was adapted to each database, as detailed in Table 1.

**Table 1:** Database search strategies

Database	Search strategy	Coverage and search date
PubMed/MEDLINE	("post-acne erythema"[Title/Abstract] OR "post acne erythema"[Title/Abstract] OR "postacne erythema"[Title/Abstract] OR "acne erythema"[Title/Abstract] OR (("post-inflammatory erythema"[Title/Abstract] OR "postinflammatory erythema"[Title/Abstract]) AND acne[Title/Abstract])) AND (random*[Title/Abstract] OR trial[Title/Abstract] OR "split-face"[Title/Abstract] OR "split face"[Title/Abstract] OR intraindividual[Title/Abstract] OR "intra-individual"[Title/Abstract])	Database inception to August 1, 2026
Scopus	TITLE-ABS-KEY("post-acne erythema" OR "post acne erythema" OR "postacne erythema" OR "acne erythema" OR (("post-inflammatory erythema" OR "post inflammatory erythema") AND acne)) AND TITLE-ABS-KEY(random* OR trial OR "split-face" OR "split face" OR intraindividual OR "intra-individual")	Database inception to August 1, 2026
Web of Science	TS=("post-acne erythema" OR "post acne erythema" OR "postacne erythema" OR "acne erythema" OR (("post-inflammatory erythema" OR "postinflammatory erythema") AND acne)) AND TS=(random* OR trial OR "split-face" OR "split face" OR intraindividual OR "intra-individual")	Database inception to August 1, 2026
Additional searching	Reference lists of included studies and relevant reviews were screened manually for randomized trials.	Completed August 1, 2026

Eligibility Criteria

Eligibility was assessed according to the Population, Intervention, Comparison, Outcome, and Study design framework. Studies were eligible when they enrolled individuals with persistent erythema following acne and evaluated an intervention using a randomized parallel-group or split-face design. Any intervention intended to improve PAE was eligible.

Comparators could include no treatment, an untreated contralateral facial side, another active intervention, or the same background treatment without the intervention of interest. Studies were required to report at least one separately extractable erythema outcome assessed using a clinician-rated scale, instrumental measurement, colorimetry, standardized photography, or image analysis. Studies evaluating multiple post-acne sequelae were eligible only when erythema findings could be extracted separately.

Patient-reported improvement, satisfaction, quality of life, procedural pain, adverse events, and recurrence or relapse were considered secondary outcomes. Non-randomized and uncontrolled studies, observational studies, case reports, case series, reviews, protocols without results, and reports without extractable randomized erythema data were excluded. The criteria are summarized in Table 2.

**Table 2:** Study eligibility criteria according to the PICOS framework PAE, Post-acne erythema.

PICOS element	Inclusion criteria	Exclusion criteria
Population	Individuals with PAE or acne-associated post-inflammatory erythema	Erythema unrelated to acne, or PAE findings that could not be extracted separately
Intervention	Any intervention intended to improve PAE	Intervention not directed at PAE
Comparator	No treatment, untreated facial side, active treatment, or background therapy alone	No randomized comparator
Outcomes	At least one extractable clinical, instrumental, colorimetric, photographic, or image-based erythema outcome	No extractable erythema outcome
Study design	Randomized parallel-group or split-face controlled trial	Non-randomized, uncontrolled, observational, review, case-series, case-report, or protocol publication without results

Study Selection

After removal of duplicates, two reviewers independently screened titles and abstracts against the eligibility criteria. A report considered potentially relevant by either reviewer underwent full-text assessment. The same reviewers independently assessed full-text reports and resolved disagreements through discussion and consensus. Reasons for full-text exclusion were documented.

Multiple reports describing the same randomized dataset were considered together to prevent duplicate inclusion. Study selection was documented using a PRISMA 2020 flow diagram [9].

Data Extraction

Two reviewers independently extracted data using a standardized form. Discrepancies were resolved by re-examining the source publication and reaching consensus. Extracted information included study design, randomized unit, blinding, sample size, participant characteristics, intervention and comparator details, treatment parameters, outcome instruments, assessment times, follow-up, attrition, and numerical results. Post-treatment values and changes from baseline were extracted as reported, together with available measures of dispersion and statistical uncertainty. These effect measures were recorded separately and were not treated as interchangeable. Event counts, proportions, ordinal categories, and comparative P values were extracted for categorical outcomes when available. Missing numerical results were not imputed.

Patient-reported outcomes, procedural pain, adverse events, and recurrence or relapse were extracted separately. Participant-level outcomes in split-face trials were not interpreted as randomized between-side effects unless side-specific results were reported. Randomized between-arm or between-side comparisons were prioritized over within-group changes. Internal inconsistencies in source reports were documented rather than resolved by assumption. When endpoint values conflicted with reported changes from baseline, the effect measure presented in the principal comparative results table was prioritized and the inconsistency was disclosed.

Risk-of-Bias Assessment

Two reviewers independently assessed risk of bias using the Cochrane Risk of Bias 2 tool [10] for the principal randomized erythema result at the main assessment time reported by each trial. Disagreements were resolved by consensus. When a prespecified primary erythema outcome was identifiable, that result was prioritized; otherwise, the principal comparative erythema result presented in the main results table was assessed. For split-face trials, facial-side randomization, within-participant analysis, missing participants, potential contralateral effects, and outcome-assessor blinding were considered.

The assessed domains were bias arising from the randomization process, bias due to deviations from intended interventions, bias due to missing outcome data, bias in measurement of the outcome, and bias in selection of the reported result. Domain-level and overall judgments were classified as low risk, some concerns, or high risk according to the RoB 2 decision algorithm. Results were summarized in a traffic-light figure.

Data Synthesis and Statistical Analysis

Meta-analysis was not performed because the trials differed substantially in interventions, comparators, randomized units, treatment schedules, outcome measures, effect metrics, and assessment times. Most interventions were represented by a single small trial, precluding a clinically interpretable pooled estimate.

Findings were synthesized narratively and organized by comparator type. Comparisons with untreated facial sides or blank controls were considered separately from active-treatment and add-on comparisons. Overlapping comparisons from the same multi-arm trial were treated as non-independent. Randomized between-arm or between-side findings were prioritized over within-group changes. Endpoint values, change scores, measures of dispersion, confidence intervals, and P values were reported as available and were not treated as interchangeable.

Patient-reported and safety outcomes were summarized descriptively because of differences in instruments, definitions, assessment times, and reporting formats. Participant-level outcomes from split-face trials were distinguished from side-specific outcomes. Statistical heterogeneity, meta-regression, funnel plots, and publication-bias testing were not performed because no meta-analysis was conducted and the number of trials was small. A formal certainty-of-evidence assessment was not conducted.

Results 

Study Selection

The database search identified 128 records. After removal of 24 duplicates, 104 records underwent title and abstract screening, of which 79 were excluded. Twenty-five full-text reports were retrieved and assessed for eligibility. Sixteen reports were excluded for predefined eligibility reasons, including non-randomized study design, an ineligible target condition, non-separable PAE outcome data, an ineligible comparator, or a duplicate or overlapping study population. Nine RCTs met the eligibility criteria and were included in the systematic review (Figure 1).

**Figure 1:**
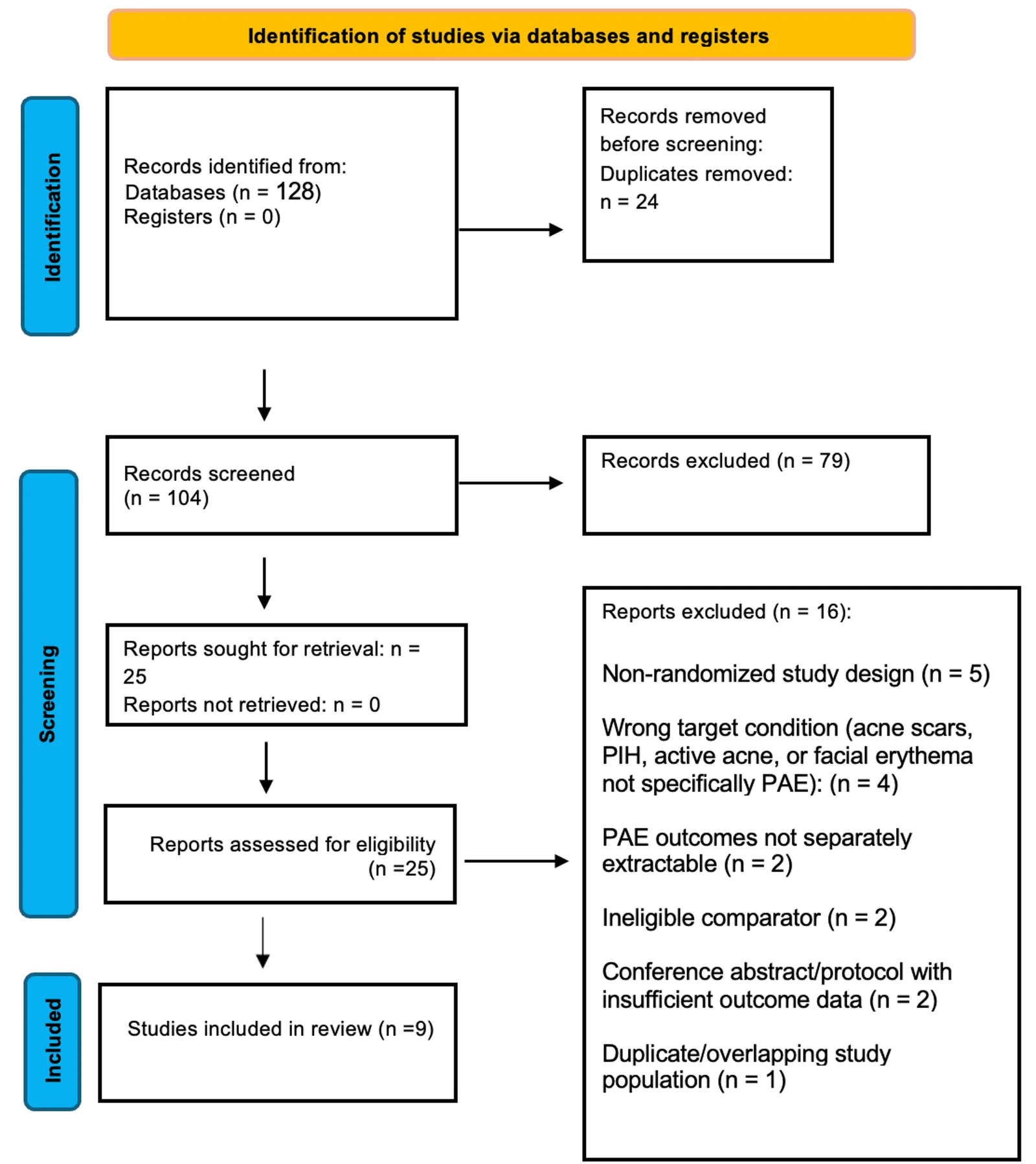
PRISMA 2020 flow diagram of the study selection process The diagram summarizes study identification, screening, eligibility assessment, and inclusion in the systematic review, adapted from the PRISMA 2020 statement [9]. PRISMA, Preferred Reporting Items for Systematic Reviews and Meta-Analyses.

Study Characteristics

Nine RCTs published between 2014 and 2026 reported sample sizes totaling 301 participants, with individual sample sizes ranging from 12 to 60 were included. Park et al. [11] and Wen et al. [12] used intraindividual split-face randomized designs. Al-Quran et al. [13] used a three-arm participant-level design, whereas Albalat et al. [14] used a two-arm parallel-group design. Yang et al. [15] and Zhang et al. [16] used intraindividual split-face randomized designs. Wanitphakdeedecha et al. [17] conducted a split-face randomized pilot trial, Mohamed et al. [18] used a two-arm parallel-group design, and Liu et al. [19] conducted a randomized, self-controlled, split-face trial. Five trials were conducted in China, two in Egypt, one in South Korea, and one in Thailand.

Blinded outcome assessment was explicitly reported by Park et al. [11], Wen et al. [12], Yang et al. [15], Wanitphakdeedecha et al. [17], and Liu et al. [19]. Al-Quran et al. [13] and Zhang et al. [16] used assessors who were not involved in treatment delivery but did not explicitly describe them as blinded. All enrolled participants were analyzed in Park et al. [11], Wen et al. [12], Yang et al. [15], Mohamed et al. [18], and Liu et al. [19], although Mohamed et al. [18] did not provide a detailed participant-flow statement. Zhang et al. [16] reported that 23 of 25 participants completed treatment, with two discontinuations attributed to acne relapse. In Wanitphakdeedecha et al. [17], 21 of 24 enrolled participants completed the trial, but the reasons for the three losses were not reported. Attrition was not clearly reported by Al-Quran et al. [13] or Albalat et al. [14].

Participants were predominantly young adults, although Wanitphakdeedecha et al. [17] reported a higher mean age of 35.0 ± 3.5 years. Sex was reported in seven trials and showed an overall female predominance. Wen et al. [12] and Liu et al. [19] did not report participant sex. Fitzpatrick skin types II to V were documented in five trials. Terminology varied across reports: Park et al. [11] used acne erythema, whereas Wen et al. [12] used post-inflammatory erythema. Reported PAE duration ranged from one month to 13 years, although duration was not consistently reported.

Interventions included fractional, vascular, picosecond, and Q-switched laser or light-based treatments [11-14,17-19], intradermal botulinum toxin type A [15], and non-cross-linked hyaluronic acid mesotherapy [16]. Albalat et al. [14] evaluated long-pulsed 1064-nm neodymium-doped yttrium aluminum garnet (Nd) laser added to low-dose isotretinoin and reported a significant baseline age difference between groups (25.13 ± 5.21 vs 20.67 ± 4.53 years; P=0.004). Comparators included active device treatments, topical brimonidine, untreated contralateral facial sides, a blank no-treatment arm, and background isotretinoin alone. Treatment exposure ranged from one to six sessions. Erythema was assessed using clinician-rated scales, instrumental erythema indices, colorimetry, photographic or image-based analysis, and patient-reported assessments. Principal randomized assessments ranged from treatment completion to three months after treatment, with longer observational follow-up in selected trials (Table 3). 

**Table 3:** Characteristics of the included randomized controlled trials (n=9) BBL, broadband light; BTX-A, botulinum toxin type A; CAT, Clinician Erythema Assessment, Area, and Telangiectasia; CEA, Clinician Erythema Assessment; CEAS, Clinician Erythema Assessment Scale; DLQI, Dermatology Life Quality Index; DPL, delicate pulsed light; EI, erythema index; GAIS, Global Aesthetic Improvement Scale; HA, hyaluronic acid; HOPSL, high-power optically pumped semiconductor laser; IPL, intense pulsed light; MI, melanin index; Nd, neodymium-doped yttrium aluminum garnet; NR, not reported; PAE, post-acne erythema; PDL, pulsed dye laser; PIE, post-inflammatory erythema; PSA, Patient Self-Assessment.

Study	Country	Design and randomized unit	Participants	Population characteristics	Intervention	Comparator	Treatment schedule	PAE/erythema assessment	Follow-up
Park et al., 2014 [11]	South Korea	Randomized split-face, evaluator-blinded pilot trial; facial-side randomization	n=12; all 12 completed	Age 20-28 years; mean 23.25 ± 3.17 years; 10 female and 2 males; bilateral acne erythema; Fitzpatrick skin type and erythema duration NR	1550-nm nonablative fractional erbium-glass laser	595-nm PDL applied to the contralateral hemiface	Three sessions at 4-week intervals	Mexameter EI; investigator-rated improvement	2 months after the final treatment
Wen et al., 2020 [12]	China	Randomized split-face, investigator-blinded trial; facial-side randomization	n=16; all 16 completed	Age, sex, and erythema duration NR; Fitzpatrick types III-V; PIE with atrophic acne scars; active acne excluded	755-nm picosecond alexandrite laser with a diffractive lens array	Untreated contralateral hemiface	Three sessions at 1-month intervals	CEAS assessed from VISIA-CR photographs by two independent blinded physicians	1 month after the final treatment
Al-Quran et al., 2023 [13]	China	Prospective three-arm randomized trial; participant-level randomization; assessment by a dermatologist not involved in treatment delivery, with blinding not explicitly stated	n=60; 20 participants per arm; attrition NR	Age 17-42 years; mean 25.95 years; 51 female and 9 males; Fitzpatrick skin type NR; PAE for at least 6 months; reported lesion duration 1-13 years	IPL with a narrow-spectrum dual-band vascular filter at 530-650 nm and 900-1200 nm	IPL with a broad-spectrum filter at 560-1200 nm or 590-1200 nm, selected according to skin color; blank no-treatment control	Four IPL sessions at 4-week intervals; no treatment in the blank-control arm	CAT composite; investigator global assessment of improvement	Approximately 1 month after treatment; acne and erythema recurrence assessed at 4 months
Albalat et al., 2024 [14]	Egypt	Randomized two-arm parallel-group trial; participant-level randomization; blinding NR	n=48; 24 participants per arm; attrition NR	Mean age 25.13 ± 5.21 vs 20.67 ± 4.53 years (P = 0.004); 9 male/15 female vs 13 male/11 female; Fitzpatrick types III-IV; PAE resistant to conventional treatment for 6 months	Long-pulsed 1064-nm Nd laser added to oral isotretinoin 10 mg/day	Oral isotretinoin 10 mg/day alone	Six Nd sessions at 2-week intervals; isotretinoin administered in both groups, with total duration NR	CEAS; ImageJ optical-density analysis; investigator-rated photographic improvement	End of the treatment course; subsequent assessment interval NR
Yang et al., 2025 [15]	China	Randomized split-face, single-blind trial with blinded outcome assessment; cheek-level randomization	n=30; all 30 analyzed	Age 18-35 years; mean 24.5 ± 4.8 years; 16 female and 14 males; bilateral PAE; Fitzpatrick skin type and PAE duration NR	Intradermal BTX-A at 1-2 U per erythematous macule; mean 18 ± 4 injection points and 18-36 U per cheek	590-nm BBL applied to the contralateral cheek	One BTX-A treatment versus three BBL sessions at 1-month intervals	EI as the primary outcome; CEA; colorimetric a* value; supportive VISIA red-area assessment	1, 2, and 3 months after treatment
Zhang et al., 2026 [16]	China	Prospective randomized split-face trial; facial-side randomization; CEAS assessment by three dermatologists not involved in treatment delivery, with blinding not explicitly stated	n=25; 23 completed; two discontinued during treatment because of acne relapse	Age 18-35 years; mean 24.0 ± 3.3 years; 19 female and 4 males among the 23 completers; Fitzpatrick types II-V; PAE duration 1-6 months	Non-cross-linked hyaluronic acid mesotherapy	Untreated contralateral hemiface	Three sessions at 4-week intervals	CEAS; VISIA red-area analysis; instrumental EI; dermoscopic assessment	Week 12, corresponding to 4 weeks after the final session; acne relapse assessed at week 24
Wanitphakdeedecha et al., 2020 [17]	Thailand	Randomized split-face controlled pilot trial; facial-side randomization; outcome assessment by two blinded dermatologists	n=24 enrolled; 21 completed; reasons for three losses NR	Mean age 35.0 ± 3.5 years; 16 female and 5 males among completers; Fitzpatrick types III-V; bilateral PAE with more than 10 lesions per facial side	577-nm HOPSL	Untreated contralateral facial side	Three sessions at 1-month intervals	Mexameter EI and MI; patient-rated and blinded dermatologist-rated overall improvement	1 month after each session, including 1 month after the third session
Mohamed et al., 2025 [18]	Egypt	Randomized two-arm parallel-group comparative trial; participant-level allocation; randomization method and blinding NR	n=60; 30 per group; all 60 included in the reported analyses; detailed participant flow NR	Mean age 22.07 ± 2.64 vs 20.93 ± 2.88 years; age range 16-27 vs 16-25 years; 22 female/8 male vs 20 female/10 male; Fitzpatrick types III-IV; mean PAE duration 9.30 ± 2.56 vs 9.67 ± 3.41 months	577-nm pro-yellow laser	Topical brimonidine tartrate 0.33% gel	Four laser sessions at 3-week intervals versus brimonidine once daily for 3 months	CEA; PSA; dermoscopic ImageJ erythema-area and optical-density analysis	End of treatment; monthly follow-up for 3 months after treatment
Liu et al., 2025 [19]	China	Prospective randomized, self-controlled, evaluator-blinded split-face trial; facial-side randomization	n=26 completed and analyzed; the methods stated that 27 eligible participants were enrolled	Adults aged 18-55 years with bilateral PAE lasting at least 3 months; sex, mean age, and Fitzpatrick skin type NR	Low-energy DPL	Low-dose Q-switched 1064-nm Nd laser applied to the contralateral facial side	Six sessions at 2-week intervals	PAE lesion count; CEA; VISIA red-zone analysis; EI; GAIS; DLQI; patient satisfaction; skin physiological measures	Assessments through week 12

Randomized Comparative Effects on Erythema

Meta-analysis was not performed because the trials differed substantially in interventions, comparators, outcome measures, effect metrics, and assessment times. Nine trials contributed 11 pairwise randomized comparisons: five against untreated or blank controls and six active-treatment or add-on comparisons. Three overlapping comparisons originated from the three-arm trial by Al-Quran et al. [13] and were recognized as non-independent comparisons from the same randomized dataset. The synthesis focused on randomized between-arm and between-side comparisons, as summarized in Table 4.

**Table 4:** Randomized comparative effects on erythema BTX-A, botulinum toxin type A; CAT, Clinician Erythema Assessment, Area, and Telangiectasia composite; CEA, Clinician Erythema Assessment; CEAS, Clinician Erythema Assessment Scale; CIELAB, Commission Internationale de l'Éclairage L*, a*, and b* color space; DPL, delicate pulsed light; EI, erythema index; HA, hyaluronic acid; HOPSL, high-power optically pumped semiconductor laser; IPL, intense pulsed light; MI, melanin index; Nd, neodymium-doped yttrium aluminum garnet; NR, not reported; PDL, pulsed dye laser; PSA, Patient Self-Assessment; SEM, standard error of the mean.

Study	Randomized comparison	Erythema outcome	Effect measure	Randomized comparative result	Statistical uncertainty	Assessment timing
Park et al., 2014 [11]	1550-nm fractional laser vs 595-nm PDL	Mexameter EI; investigator and patient assessments	Post-treatment EI and improvement ratings from baseline	EI 320.33 ± 8.47 vs 329.02 ± 8.74. Investigators score 3.42 ± 0.67 vs 3.33 ± 0.65. Patient-rated good or excellent improvement 91.7% vs 75.0%.	EI difference not statistically significant; comparative P values for subjective outcomes NR	2 months after the final session
Wen et al., 2020 [12]	755-nm picosecond alexandrite laser vs untreated hemiface	CEAS	Post-treatment values	1.75 ± 0.45 vs 3.19 ± 0.54, favoring laser treatment	P < 0.05	1 month after the final session
Al-Quran et al., 2023 [13]	Narrow-spectrum vascular IPL vs blank control	CEA; affected area; total CAT; investigator-rated improvement	Post-treatment values and categorical distribution	CEA 1 vs 3; area 1 vs 2; total CAT 1 vs 6. Published improvement categories in the vascular group were 10/20 excellent, 8/20 labelled "good," 1/20 marked, and 1/20 fair, compared with 0/20 excellent, 0/20 good, 7/20 marked, and 13/20 fair in the blank-control group.	Investigator-rated distribution P < 0.001; CAT outcomes showed post hoc separation	Approximately 1 month after treatment
Al-Quran et al., 2023 [13]	Broad-spectrum IPL vs blank control	CEA; affected area; total CAT; investigator-rated improvement	Post-treatment values and categorical distribution	CEA 2.5 vs 3; area 2 vs 2; total CAT 6 vs 6. Published improvement categories were 1/20 excellent, 2/20 labelled "good," 8/20 marked, and 9/20 fair with broad-spectrum IPL, compared with 0/20, 0/20, 7/20, and 13/20, respectively, in the blank-control group.	Investigator-rated distribution P = 0.174; CAT outcomes shared post hoc grouping	Approximately 1 month after treatment
Al-Quran et al., 2023 [13]	Narrow-spectrum vascular IPL vs broad-spectrum IPL	CEA; affected area; total CAT; investigator-rated improvement	Post-treatment values and categorical distribution	CEA 1 vs 2.5; area 1 vs 2; total CAT 1 vs 6. The published investigator-rated distribution favored vascular IPL, but one category was inconsistent with the prespecified scale.	Investigator-rated distribution P < 0.001; CAT outcomes showed post hoc separation	Approximately 1 month after treatment
Albalat et al., 2024 [14]	Low-dose isotretinoin plus 1064-nm Nd vs low-dose isotretinoin alone	CEAS; ImageJ optical density; investigator-rated improvement	Post-treatment values and categorical response	CEAS 1.29 ± 0.98 vs 1.96 ± 0.93; optical density 0.278 ± 0.112 vs 0.365 ± 0.021; excellent or good response 15/24 vs 5/24, favoring add-on Nd	P = 0.03, P = 0.04, and P = 0.03, respectively	End of the six-session treatment course
Yang et al., 2025 [15]	Intradermal BTX-A vs broadband light	CEA; EI; CIELAB redness coordinate	Change from baseline	CEA changes were -0.80 vs -0.50 at month 1, -1.40 vs -1.03 at month 2, and -1.63 vs -1.47 at month 3. EI and CIELAB redness reductions favored BTX-A at month 1; only the CIELAB redness difference remained significant at month 3.	CEA: P = 0.032, 0.014, and 0.343; EI: P = 0.001, 0.647, and 0.595; CIELAB redness: P < 0.001, 0.429, and 0.019 at months 1, 2, and 3, respectively	Months 1, 2, and 3
Zhang et al., 2026 [16]	Non-cross-linked HA mesotherapy vs untreated hemiface	CEAS; VISIA red area; EI	Post-treatment values reported as mean ± dispersion; the article describes normally distributed tabulated data as mean ± SEM, although the individual outcome tables do not label the dispersion measure	CEAS 1.8 ± 0.6 vs 2.3 ± 0.9; red area 27.69 ± 5.89 vs 31.12 ± 4.77; EI 307 ± 53 vs 334 ± 57, favoring mesotherapy	P = 0.042, P = 0.035, and P = 0.041, respectively	Week 12, 4 weeks after the final session
Wanitphakdeedecha et al., 2020 [17]	577-nm HOPSL vs untreated hemiface	Mexameter EI and MI; patient-rated and blinded dermatologist-rated improvement	Serial between-side comparisons	EI reduction favored the laser-treated side 1 month after the second treatment. Patient-rated improvement scores were 2.1 vs 1.2, 2.3 vs 1.3, and 2.3 vs 1.3 after sessions 1, 2, and 3. Blinded dermatologist scores were 1.9 vs 1.7, 2.6 vs 2.5, and 3.1 vs 3.1. No significant comparative MI difference was reported.	EI: P = 0.007 after the second treatment. Patient ratings: P = 0.002, 0.001, and <0.001. Blinded dermatologist ratings: P = 0.519, 0.776, and 0.822.	1 month after each treatment session
Mohamed et al., 2025 [18]	577-nm pro-yellow laser vs topical brimonidine tartrate 0.33%	CEA; PSA; ImageJ erythema-area percentage and optical density; investigator global assessment	Post-treatment values and categorical distributions	Erythema-area percentage 10.90 ± 6.13 vs 17.13 ± 5.69 and optical density 2.89 ± 0.70 vs 3.80 ± 0.66, favoring laser. The published CEA and PSA tables both reported clear or almost-clear status in 28/30 vs 19/30; the identical distributions may reflect duplicated source reporting. Investigator-rated excellent improvement occurred in 18/30 vs 2/30.	P < 0.001 for erythema area, optical density, the reported CEA and PSA distributions, and investigator global-assessment distribution	End of the 3-month treatment period
Liu et al., 2025 [19]	Low-energy DPL vs Q-switched 1064-nm Nd laser	PAE lesion count; CEA; EI; VISIA red-zone area	Serial change from baseline and between-side comparisons	Both sides improved. DPL produced a greater reduction in PAE lesion count that remained significant at week 12 (16.500 ± 2.437 vs 17.654 ± 2.560). DPL also produced a lower CEA score at week 8, but the between-side differences in CEA, EI, and VISIA red-zone area were not statistically significant at week 12.	PAE lesion count: P = 0.015 at week 12; CEA: P = 0.043 at week 8; between-side CEA, EI, and red-zone comparisons: P > 0.05 at week 12	Weeks 4, 8, and 12

Comparisons With Untreated or Blank Controls

Five comparisons from four trials involved untreated or blank controls: Wen et al. [12], Al-Quran et al. [13], Zhang et al. [16], and Wanitphakdeedecha et al. [17]. None of the nine included trials used a sham intervention.

Wen et al. [12] reported a lower clinician-rated erythema score following picosecond alexandrite laser treatment than on the untreated contralateral hemiface one month after the final session. Zhang et al. [16] similarly reported lower post-treatment Clinician Erythema Assessment Scale (CEAS), VISIA red-area, and instrumental erythema-index values with non-cross-linked hyaluronic acid mesotherapy than on the untreated hemiface at week 12.

In the three-arm trial by Al-Quran et al. [13], narrow-spectrum vascular IPL was favored over the blank control for post-treatment Clinician Erythema Assessment (CEA), affected area, total Clinician Erythema Assessment, Area, and Telangiectasia (CAT) score, and investigator-rated improvement. Broad-spectrum intense pulsed light (IPL) did not show significant separation from the blank control. The telangiectasia component was zero in all participants and therefore did not independently contribute to between-group differences. Baseline affected-area and total CAT scores differed between groups, and the reported endpoint comparisons were not adjusted for these differences. In addition, the published investigator-assessment table included a category labelled "good," although the prespecified scale comprised poor, fair, marked, and excellent improvement. Categories were therefore retained as reported and were not combined.

Wanitphakdeedecha et al. [17] found that the reduction in instrumental erythema index (EI) favored the 577-nm high-power optically pumped semiconductor laser (HOPSL)-treated side over the untreated side one month after the second session (P=0.007). Patient-rated improvement favored the treated side after each session, whereas improvement ratings from two blinded dermatologists did not differ significantly between sides at any assessment. Both facial sides showed reductions in EI by the final assessment, and no significant between-side difference in melanin index (MI) was reported.

The untreated sides in Wen et al. [12] and Zhang et al. [16] showed comparatively little change, whereas the untreated side in Wanitphakdeedecha et al. [17] showed a significant within-side reduction in EI by the final assessment. These findings were interpreted only as concurrent randomized control observations and not as evidence of the natural course of PAE.

Active-Treatment and Add-On Comparisons

Six randomized comparisons evaluated active treatments or add-on therapy: fractional laser versus pulsed dye laser [11], narrow-spectrum versus broad-spectrum IPL [13], long-pulsed neodymium-doped yttrium aluminum garnet (Nd) laser plus low-dose isotretinoin versus isotretinoin alone [14], intradermal botulinum toxin type A versus broadband light [15], 577-nm pro-yellow laser versus topical brimonidine [18], and low-energy delicate pulsed light (DPL) versus Q-switched 1064-nm Nd laser [19].

Park et al. [11] reported reductions in instrumental erythema with both fractional laser and pulsed dye laser, without a statistically significant between-side difference. Investigator and patient assessments numerically favored fractional laser, but formal between-side significance tests were not reported for these subjective outcomes.

Narrow-spectrum vascular IPL was favored over broad-spectrum IPL for post-treatment CEA, affected area, total CAT score, and investigator-rated improvement [13]. Albalat et al. [14] reported lower post-treatment CEAS and ImageJ optical-density values and a more favorable investigator-rated response distribution when long-pulsed Nd laser was added to low-dose isotretinoin. Because both groups received isotretinoin, this comparison estimates the incremental effect of adding Nd laser rather than the isolated efficacy of the laser.

Yang et al. [15] reported greater reductions with BTX-A than with broadband light at month one in CEA score, EI, and the Commission Internationale de l'Éclairage L*, a*, and b* color space (CIELAB) redness coordinate. The between-side difference remained statistically significant for CEA at month two and for the CIELAB redness coordinate at month three, whereas the remaining later comparisons were not statistically significant. The article contained an internal inconsistency between the post-treatment CEA values reported in the narrative and the changes from baseline reported in its principal results table. The change estimates were used because they were internally consistent with the baseline values and the reported comparative analysis.

Mohamed et al. [18] reported lower post-treatment erythema-area percentage and optical density with 577-nm pro-yellow laser than with topical brimonidine. Investigator global-assessment findings also favored the laser group. The published CEA and Patient Self-Assessment (PSA) tables contained identical categorical distributions and identical comparative statistics; these outcomes were therefore retained as reported but interpreted cautiously because independent verification of the two distributions was not possible. Dermoscopic images were described as having been selected haphazardly for ImageJ analysis.

Liu et al. [19] reported improvement with both low-energy DPL and Q-switched 1064-nm Nd laser. DPL produced a greater reduction in PAE lesion count that remained statistically significant at week 12 (16.500 ± 2.437 vs 17.654 ± 2.560; P=0.015). DPL also produced a lower CEA score at week eight, but this difference was no longer statistically significant at week 12. At the final assessment, reductions in EI and VISIA red-zone area were significant within both treated sides, without significant between-side differences. These findings suggest a sustained comparative effect on lesion count but do not establish overall superiority across erythema outcomes.

Patient-Reported Outcomes and Safety

Patient-reported outcomes included patient-rated improvement, satisfaction, procedural pain, the Cardiff Acne Disability Index (CADI), Dermatology Life Quality Index (DLQI), and PSA. Differences in instruments, units of analysis, and assessment times precluded quantitative synthesis.

Park et al. [11] reported good or excellent patient-rated improvement in 91.7% of fractional laser-treated sides and 75.0% of PDL-treated sides at two months, although no formal between-side significance test was reported. Wen et al. [12] reported participant-level satisfaction at one month, with 62.5% satisfied and 12.5% very satisfied. Because satisfaction was not reported separately for each randomized facial side, it could not be interpreted as a randomized treatment effect.

Al-Quran et al. [13] reported a lower post-treatment CADI with vascular-filter IPL than with broad-spectrum IPL or no treatment. The narrative stated satisfaction rates of 95%, 40%, and 10% in the vascular-filter, broad-spectrum, and untreated groups, respectively. However, applying the study's stated satisfaction definition to the category counts produced the reverse pattern. The satisfaction findings were therefore considered internally inconsistent and were not interpreted quantitatively.

Yang et al. [15] reported greater procedural pain with botulinum toxin type A (BTX-A) than with broadband light (BBL), with visual analog scale (VAS) scores of 5.0 and 3.1, respectively (P<0.001). Side-specific satisfaction did not differ significantly at three months (P=0.079). Zhang et al. [16] reported greater side-specific PSA improvement with mesotherapy (P<0.001). The same report described a reduction in DLQI from 13.6 ± 3.3 at baseline to 9.3 ± 3.1 (P<0.001). Because DLQI was measured at the participant level in a split-face trial, it could not be attributed to either randomized hemiface. The source also referred to this result as occurring in the "control group," creating additional uncertainty in its labeling.

In Wanitphakdeedecha et al. [17], patient-rated improvement favored the 577-nm HOPSL-treated side after each session. In contrast, ratings from two blinded dermatologists did not differ significantly between sides. The divergence may reflect the absence of participant blinding and the subjective nature of patient-rated improvement.

Mohamed et al. [18] reported a post-treatment PSA distribution favoring 577-nm pro-yellow laser over topical brimonidine. However, the published PSA table was identical to the CEA table in every category and comparative statistic. The PSA findings were therefore retained as reported but were not treated as independently verified evidence of patient-perceived benefit.

Liu et al. [19] reported overall patient satisfaction of 92.31%, with the distribution of ratings favoring the DPL-treated side. Global Aesthetic Improvement Scale (GAIS) improved on both treated sides without a significant between-side difference at the reported later assessments. DLQI also improved significantly over the study period, but because DLQI was measured at the participant level in a split-face design, this improvement could not be attributed separately to DPL or Q-switched Nd treatment.

Adverse events were generally mild and transient, but reporting was incomplete and follow-up was insufficient to establish long-term safety. Park et al. [11] reported stinging, redness, and scaling that resolved within one week. Wen et al. [12] reported mild erythema and edema resolving within 24 hours. Al-Quran et al. [13] reported inconsistent frequencies of post-treatment redness and flushing. One section described redness in all participants treated with broad-spectrum IPL and flushing in 5/20 participants treated with vascular-filter IPL, whereas another stated that flushing occurred in all IPL-treated participants. Pigmentation occurred in 2/20 participants and blistering in 3/20 following broad-spectrum IPL, with blisters lasting approximately four days.

Albalat et al. [14] described mild procedural discomfort and temporary facial redness lasting a few hours. Yang et al. [15] prespecified adverse-event assessment but did not report frequencies for non-pain events. Zhang et al. [16] reported mild diffuse facial erythema in all participants, generally resolving within one to three days, and no serious adverse events.

Wanitphakdeedecha et al. [17] reported mild discomfort during treatment and transient facial erythema lasting approximately 30 minutes, with no additional adverse events described. Mohamed et al. [18] reported only minor adverse effects, with no significant difference in overall incidence between treatment groups. Irritation and dryness were more frequently reported with brimonidine, whereas mild erythema occurred in both groups. Liu et al. [19] reported greater intraprocedural pain with DPL than with Q-switched Nd laser. Burning, transient erythema, and swelling occurred on both treated sides, with no significant between-side differences. No hyperpigmentation or hypopigmentation was observed.

Longer-term reporting principally concerned acne relapse rather than recurrence of PAE. Al-Quran et al. [13] reported acne relapse in 0/20, 8/20, and 3/20 participants in the vascular-filter, broad-spectrum, and untreated groups, respectively. In Zhang et al. [16], two participants discontinued treatment because of acne relapse, and one of the 23 completers experienced an additional relapse at week 24. Mohamed et al. [18] reported no recurrence of erythema in either treatment group during three months of post-treatment follow-up. The acne-relapse findings from Al-Quran et al. [13] and Zhang et al. [16] should not be interpreted as direct evidence concerning recurrence of PAE. Patient-reported outcomes, safety findings, and post-treatment observations are summarized in Table 5.

**Table 5:** Patient-reported outcomes, safety, and post-treatment observations BBL, broadband light; BTX-A, botulinum toxin type A; CADI, Cardiff Acne Disability Index; CEA, Clinician Erythema Assessment; DLQI, Dermatology Life Quality Index; DPL, delicate pulsed light; GAIS, Global Aesthetic Improvement Scale; HOPSL, high-power optically pumped semiconductor laser; IPL, intense pulsed light; Nd, neodymium-doped yttrium aluminum garnet; NR, not reported; PAE, post-acne erythema; PDL, pulsed dye laser; PSA, Patient Self-Assessment; VAS, visual analog scale.

Study	Patient-reported and quality-of-life outcomes	Procedural pain or discomfort	Adverse events and resolution	Post-treatment observations
Park et al., 2014 [11]	Good or excellent patient-rated improvement at 2 months: 91.7% with fractional laser vs 75.0% with PDL; formal between-side significance test NR	NR	Stinging, redness, and scaling; resolved within 1 week	NR
Wen et al., 2020 [12]	Participant-level satisfaction at 1 month: 62.5% satisfied and 12.5% very satisfied; not interpretable as a randomized between-side effect	VAS median 2.3, range 2-4, on the treated side	Mild erythema and edema; resolved within 24 hours	NR
Al-Quran et al., 2023 [13]	Post-treatment CADI: median 1 with vascular-filter IPL vs 10 with broad-spectrum IPL and 10 with no treatment. Satisfaction narrative and tabulated categories were internally contradictory and were not interpreted quantitatively.	Pain described as tolerated; no formal scale	Frequencies of redness and flushing were internally inconsistent. Pigmentation occurred in 2/20 participants and blistering in 3/20 following broad-spectrum IPL; blisters lasted approximately 4 days.	Acne relapse at 4 months: 0/20 with vascular-filter IPL, 8/20 with broad-spectrum IPL, and 3/20 with no treatment (P = 0.004); PAE recurrence not quantified
Albalat et al., 2024 [14]	NR	Mild discomfort during laser treatment	Temporary facial redness; resolved within a few hours	NR
Yang et al., 2025 [15]	Side-specific satisfaction did not differ significantly between BTX-A and BBL at 3 months (P = 0.079)	VAS 5.0 with BTX-A vs 3.1 with BBL (P < 0.001)	Frequencies of non-pain adverse events NR	NR
Zhang et al., 2026 [16]	Side-specific PSA favored mesotherapy (P < 0.001). Participant-level DLQI decreased from 13.6 ± 3.3 to 9.3 ± 3.1 (P < 0.001) but was not attributable to either randomized hemiface.	NR	All participants experienced mild diffuse facial erythema, generally resolving within 1-3 days. No serious adverse events were reported.	Two participants discontinued treatment because of acne relapse; one additional relapse occurred among 23 completers at week 24. PAE recurrence NR.
Wanitphakdeedecha et al., 2020 [17]	Patient-rated improvement scores favored HOPSL after sessions 1, 2, and 3: 2.1 vs 1.2, 2.3 vs 1.3, and 2.3 vs 1.3 (P = 0.002, 0.001, and <0.001). Blinded dermatologist ratings did not differ significantly between sides.	Mild discomfort during treatment	Transient facial erythema lasting approximately 30 minutes; no additional adverse events were described.	Follow-up ended 1 month after the third session; longer-term PAE recurrence NR
Mohamed et al., 2025 [18]	Published PSA distribution favored pro-yellow laser (P < 0.001), but the PSA and CEA tables contained identical distributions and statistics and may reflect duplicated source reporting.	Formal pain assessment NR	Brimonidine: mild erythema 8/30, irritation 12/30, and dryness 4/30. Pro-yellow laser: mild erythema 10/30 and irritation 6/30. Adverse effects were described as minor and resolving gradually; overall incidence did not differ significantly.	No recurrence of erythema was detected in either group during 3 months of post-treatment follow-up.
Liu et al., 2025 [19]	Overall patient satisfaction was reported as 92.31%, with the distribution of ratings favoring DPL. GAIS improved on both sides without a significant later between-side difference. Participant-level DLQI improved during follow-up but was not attributable to either randomized facial side.	Median intraprocedural VAS 3.0 with DPL vs 1.0 with Q-switched Nd (P < 0.05); immediate post-treatment VAS 0 vs 0 (P = 0.071)	Burning: 10 vs 5; transient erythema: 15 vs 13; swelling: 3 vs 1 with DPL and Q-switched Nd, respectively. Between-side differences were not significant. No hyperpigmentation or hypopigmentation was observed.	Follow-up through week 12; long-term PAE recurrence NR

Risk-of-Bias Assessment

Overall risk-of-bias judgments were some concerns for five trials [11,12,15,16,19] and high risk for four trials [13,14,17,18]. Bias arising from the randomization process was judged high in Al-Quran et al. [13] and Albalat et al. [14] because randomization and allocation concealment were insufficiently described and statistically significant baseline imbalances were present. Deviations from intended interventions raised some concerns in the open parallel-group trials by Al-Quran et al. [13] and Mohamed et al. [18], which provided insufficient information on co-interventions or treatment adherence. Missing outcome data raised some concerns in Zhang et al. [16], Wanitphakdeedecha et al. [17], and Liu et al. [19].

Measurement of the outcome was judged at high risk in Mohamed et al. [18] because assessor blinding was not reported and dermoscopic images were selected haphazardly for analysis. Selection of the reported result raised at least some concerns in every trial because prospective outcome and analysis prespecification could not be verified. Wanitphakdeedecha et al. [17] was judged at high risk in this domain because a significant interim between-side erythema-index comparison was reported without an equivalent final estimate, while favorable unblinded patient ratings were emphasized despite null blinded-assessor comparisons. Overall risk-of-bias judgments are shown in Figure 2.

**Figure 2:**
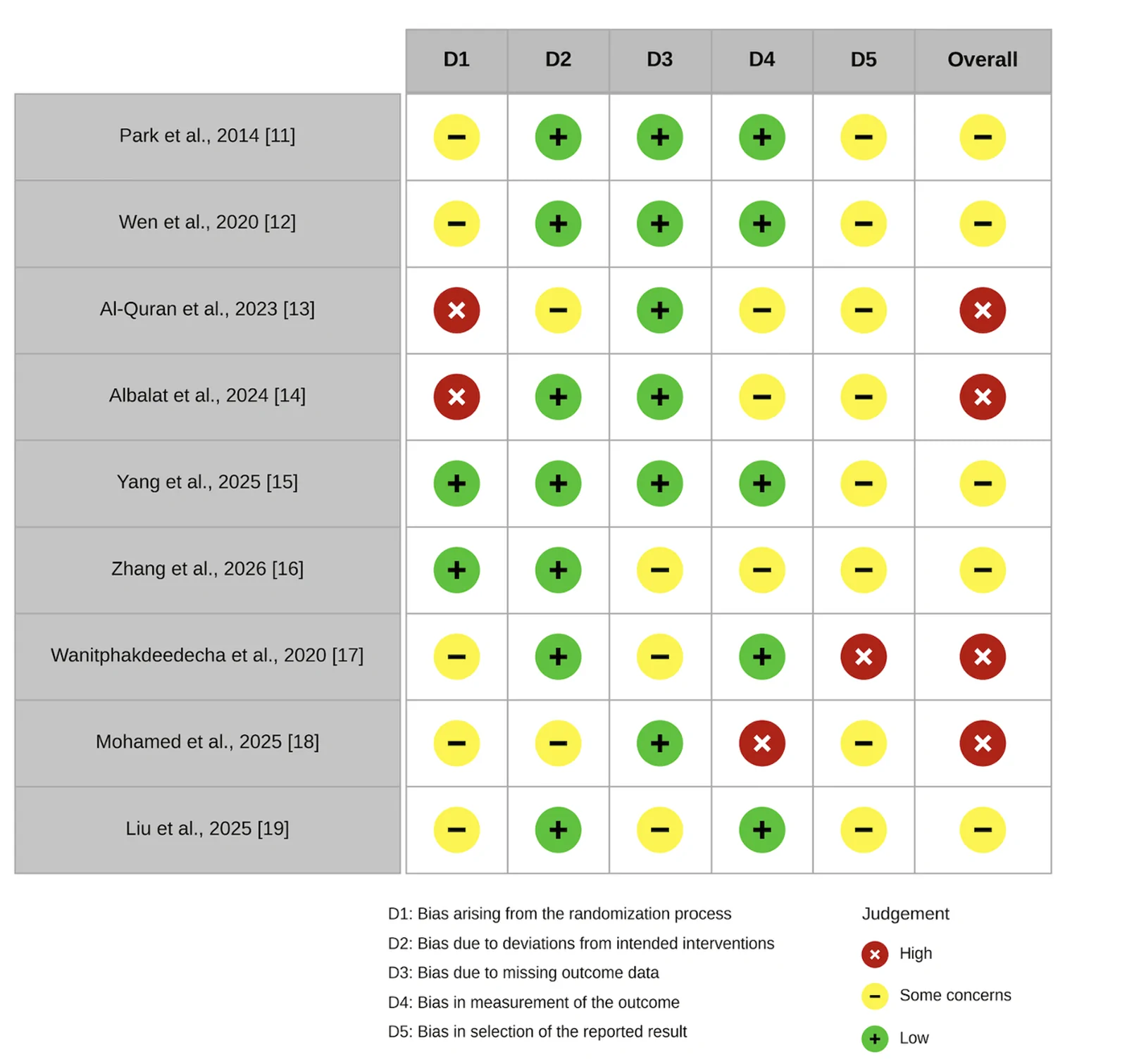
Risk-of-bias assessment of the included randomized controlled trials using the Cochrane Risk of Bias 2 tool D1, bias arising from the randomization process; D2, bias due to deviations from intended interventions; D3, bias due to missing outcome data; D4, bias in measurement of the outcome; D5, bias in selection of the reported result. Green indicates low risk of bias, yellow indicates some concerns, and red indicates high risk of bias. Risk of bias was assessed using the Cochrane Risk of Bias 2 (RoB 2) tool [10].

Discussion

Nine randomized trials contributing 11 pairwise comparisons identified favorable effects but did not support a treatment ranking or standard of care. Against untreated or blank controls, picosecond alexandrite laser, non-cross-linked hyaluronic acid mesotherapy, and narrow-spectrum vascular IPL reduced erythema, whereas broad-spectrum IPL did not differ from blank control [12,13,16]. The 577-nm HOPSL produced an interim instrumental benefit, but no equivalent final between-side estimate was reported, and blinded dermatologist ratings did not differ between sides [17]. In active-treatment and add-on comparisons, narrow-spectrum IPL was favored over broad-spectrum IPL, adding Nd laser was favored over isotretinoin alone, and pro-yellow laser was favored over topical brimonidine [13,14,18]. BTX-A was favored over broadband light at early assessments, while fractional laser and pulsed dye laser did not differ significantly [11,15]. DPL produced a sustained reduction in lesion count relative to Q-switched 1064-nm Nd laser, but final CEA, EI, and red-zone comparisons were not significant [19]. Each comparison arose from a single small trial.

These device findings represent separate randomized comparisons rather than a therapeutic hierarchy. Selective photothermolysis depends on wavelength, pulse duration, and fluence because vascular targeting requires absorption by the intended chromophore and exposure compatible with its thermal relaxation time [20]. This principle may help explain the narrow-spectrum IPL result, but that trial had unadjusted baseline differences and inconsistent investigator-rated and patient-reported data. Treatment schedules, penetration depth, mechanisms, and baseline severity also differed. The pro-yellow laser comparison expanded the evidence to an active topical comparator, but identical CEA and PSA tables and haphazard selection of dermoscopic images substantially weaken confidence in its reported advantage.

Because both groups in the add-on trial received low-dose isotretinoin, its result estimates the incremental effect of Ndlaser rather than isolated laser efficacy. The BTX-A advantage was not consistent across outcomes or assessments, and BTX-A caused greater procedural pain. Loss of statistical significance does not establish equivalence. Participant-level satisfaction and quality-of-life outcomes in split-face trials cannot be attributed to either randomized facial side [21]. This limitation applies particularly to DLQI findings reported by Zhang et al. and Liu et al. [16,19].

Strengths include restriction to randomized evidence, separation of comparative effects from within-group improvement, and consideration of clinical, instrumental, patient-reported, and safety outcomes. Nevertheless, five trials had some concerns and four had high overall risk of bias. Limitations included small samples, predominantly single-center designs, incomplete blinding, unclear allocation concealment, baseline imbalance, attrition, unavailable prespecified analyses, and internal reporting inconsistencies. The contrast between favorable unblinded patient ratings and null blinded assessments in the HOPSL trial illustrates the potential influence of measurement conditions [17].

Outcome heterogeneity further limited interpretation because erythema measurements obtained using different instruments are not interchangeable [22]. Although the CEA has demonstrated reliability in rosacea, its measurement properties in PAE remain uncertain [23]. Heterogeneity precluded a meaningful pooled estimate, and nine trials were insufficient for formal assessment of publication bias. No validated threshold established whether reported differences were clinically important. Adverse events were generally mild and transient, but pigmentation and blistering occurred with one broad-spectrum IPL protocol. Small samples and short follow-up preclude firm conclusions about uncommon harms, long-term safety, or PAE recurrence.

The evidence may inform individualized decisions but does not establish a preferred intervention. Selection should consider erythema characteristics, skin phototype, active acne, treatment burden, pain, cost, access, and patient preference. Skin phototype is important because darker skin may carry a greater risk of treatment-related dyspigmentation [24]. Future trials should be adequately powered and prospectively registered, with concealed allocation, blinded assessment, standardized PAE definitions, validated measures, prespecified primary time points, comparative estimates with confidence intervals, treatment-attributable patient-reported outcomes, longer follow-up, and systematic reporting of recurrence and adverse events.

## Conclusions

Overall, the available randomized evidence suggests that several interventions may improve post-acne erythema, although the certainty and comparability of these findings remain limited. Randomized trials identified potentially favorable effects with picosecond alexandrite laser, narrow-spectrum vascular IPL, non-cross-linked hyaluronic acid mesotherapy, intradermal botulinum toxin type A, and Nd added to low-dose isotretinoin. A 577-nm HOPSL produced an interim instrumental benefit without an equivalent final between-side estimate, while blinded dermatologist ratings did not differ between sides. Pro-yellow laser was favored over topical brimonidine, although reporting and outcome-measurement limitations reduced confidence in this finding. DPL produced a sustained reduction in PAE lesion count relative to Q-switched 1064-nm Nd laser, but final CEA, EI, and red-zone comparisons did not demonstrate overall superiority. These findings arose from small, heterogeneous trials using different comparators, treatment schedules, outcome measures, and assessment times. Five trials had some concerns, and four had high overall risk of bias. The evidence therefore does not establish a preferred intervention or definitive treatment hierarchy. Adverse events were generally mild and transient, but the available samples and follow-up periods were insufficient to determine uncommon harms, long-term safety, or PAE recurrence. Treatment decisions should be individualized. Larger, adequately powered, prospectively registered trials using blinded assessment, standardized outcomes, and longer follow-up are required.
